# Consensus on the Structure and Content of Birth Plans: A Modified Delphi Study

**DOI:** 10.1111/hex.70124

**Published:** 2024-12-18

**Authors:** Françoise Vendittelli, Lucie Adalid, Violaine Peyronnet, Sophie Guillaume, Nathalie Piquée, Aurore Viard‐Cretat, Catherine Crenn‐Hébert, Olivier Rivière, Candy Guiguet‐Auclair, Study Group, Claire Audouin, Françoise Molenat, Estelle Morau, Sylvie Le Roux, Laurent Gerbaud, Cyril Huissoud

**Affiliations:** ^1^ Université Clermont Auvergne, Clermont Auvergne INP, CHU Clermont‐Ferrand, CNRS, Institut Pascal Clermont‐Ferrand France; ^2^ Association des Utilisateurs de Dossiers Informatisés en Pédiatrie Obstétrique et Gynécologie (Audipog) Université Claude Bernard Lyon 1 Lyon France; ^3^ Collectif Inter‐Associatif Autour de la Naissance (CIANE) Paris France; ^4^ Hôpital Louis Mourier, APHP Colombes France; ^5^ Collège National des Gynécologues Obstétriciens Français (CNGOF) Paris France; ^6^ Collège National des Sages‐Femmes de France (CNSF) Paris France; ^7^ Société Française de Médecine Périnatale (SFMP) Paris France; ^8^ Centre Hospitalier Universitaire de Lyon Lyon France; ^9^ Montpellier France; ^10^ Collège d'Anesthésie‐Réanimation en Obstétrique (CARO) Lyon France; ^11^ Association Nationale des Sages‐Femmes Coordinatrices (ANSFC) Rouen France

**Keywords:** childbirth, communication, consensus, delivery, Delphi method, pregnancy, prenatal care

## Abstract

**Background:**

Few pregnant women in France wrote birth plans as in many other countries. The literature stresses the heterogeneity of birth plan content, which limits the utility of assessing the effects of birth plans on women's experience of childbirth. This study aimed to obtain a French national consensus on the structure and content of birth plans.

**Methods:**

A multidisciplinary steering committee was established. An electronic modified Delphi study was conducted to develop a structure and content for birth plans between November 2022 and June 2023. During three Delphi consensus rounds, panellists, including perinatal health care professionals and user representatives, were asked to rate individually and independently each proposed section and subsection formulation of the birth plan for its appropriateness. An external board assessed the understandability of the final birth plan's preamble and content.

**Results:**

The steering committee proposed 103 formulations corresponding to items to be covered in a birth plan, categorized into 8 sections and 30 subsections, for evaluation in the Delphi rounds. The first round was completed by 42 panellists (mainly midwives), the second by 39, and the third by 36. Finally, the steering committee approved the final components of the structured birth plan in 8 sections and 19 subsections, after its reviewing by the 21 members of the external board.

**Conclusion:**

A French national Delphi process, after three rounds and validation by an external board, made it possible to reach a consensus on the structure and content of a birth plan in 8 sections and 19 subsections.

**Patient or Public Contribution:**

User representatives were included as experts in the Delphi rounds, and in the external board to approve the final version of the structured birth plan.

## 1 Introduction

Natural childbirth advocates in the 1980s introduced the first birth plans in response to the sense of loss of agency pregnant women felt in the birth process [1, 2, 3, 4]. The purpose of a birth plan is to facilitate communication between pregnant women and perinatal health care providers and encourage informed decision‐making. Criticism of overly medicalized approaches to childbirth led the World Health Organization to classify birth plans in the top category of recommended practices for making pregnancy safer [5, 6].

Birth plans are now fairly common in modern obstetrics, no longer restricted to midwifery practices. Childbirth education is supported by many Western professional societies of midwives, obstetricians and gynaecologists, and paediatricians, as well as health departments [7, 8, 9, 10, 11].

Since 2005, French regulations have encouraged early prenatal interviews for pregnancy‐related risk screening (compulsory since 2020) and prenatal parenthood education [12, 13]. Both are opportunities to explain birth plans to parents and work with them to phrase them precisely [14, 15, 16]. French health insurance is required to cover eight prenatal parenthood preparation sessions of at least 45 min, including the individual or couple prenatal interview [15]. Nonetheless, in 2021, only 10.2% of women giving birth in French hospitals had a written birth plan [17].

The pregnant woman, with or without the partner, can design a written personalized birth plan or follow a suggested template from the maternity ward or perinatal network. The parents can also inform the care provider orally about their preferences. The research literature contains a variety of formats and templates. Articles sometimes fail to describe interventions adequately, and the method for obtaining local consensus is often unclear [18, 19, 20, 21, 22, 23]. Only a few countries, such as Scotland, use a birth plan endorsed at the national level [24, 25, 26], and again, the method used to obtain a national consensus remains murky [25].

Some birth plans are restrictive standardized questionnaires with closed answers that fail to: educate and empower women, encourage them to share decision‐making, facilitate communication about expectations or develop trust between women (couples) and the perinatal professionals.

One way to improve the use of birth plans by pregnant women and perinatal professionals, particularly midwives, is to develop a national consensus about the items to be covered. This study aimed to obtain a French national consensus on the structure and content of birth plans, using a modified Delphi study.

## 2 Methods

### 2.1 Design

**Figure 1:**
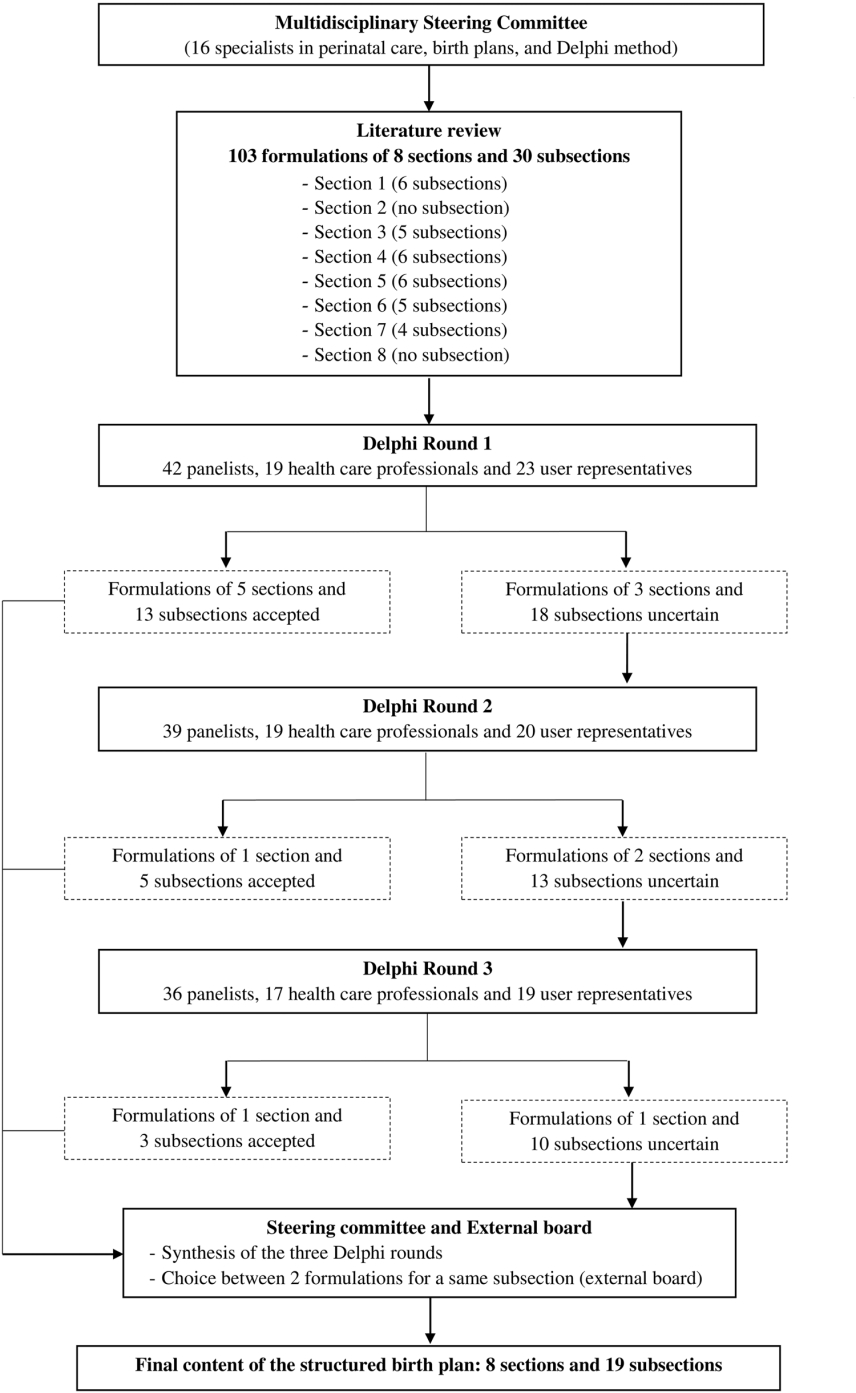
The modified Delphi process for the selection of the structured content of the birth plan.

From November 2022 through June 2023, we conducted an electronically modified RAND/University of California at Los Angeles (UCLA) appropriateness method Delphi study to develop the structure and contents of a birth plan template [27]. The RAND/UCLA process had four phases (Figure 1).

The first phase was the constitution of a French multidisciplinary steering committee, chaired by a representative of the Inter‐Associative Collective on Childbirth (CIANE), a collective of French associations concerned with issues relating to pregnancy, birth, and the first days of life, accredited to represent users in the French health care system. The steering committee was composed of 16 members: three methodological experts in Delphi methods, three scientific experts in birth plans, and nine vice‐presidents, one designated by the CIANE and one by each of the following learned societies: the National Association of Coordinating Midwives (ANSFC), the Association of Users of Computerized Records in Pediatrics, Obstetrics and Gynecology (AUDIPOG), the Obstetric Anaesthesia and Intensive Care College (CARO), the National College of French Gynaecologists and Obstetricians (CNGOF), the National College of French Midwives (CNSF), the French Society of Perinatal Medicine (SFMP), the French‐speaking Association for the study of puerperal and perinatal psychiatric pathologies (Société Marcé), the World Association for Infant Mental Health (WAIMH‐France).

In the second phase, the steering committee reviewed the literature to identify components for birth plans, wrote the first Delphi questionnaire, and then reviewed other Delphi process questionnaires.

The third phase consisted of three Delphi consensus rounds in which an expert panel rated potential components of a birth plan [28, 29, 30].

In the fourth and final phase, both an external board, composed of health care professionals and user representatives designated by the participating learned societies and the CIANE, and the steering committee separately approved the French practice advisery explaining the final structured birth plan to perinatal caregivers and users.

We modified the RAND/UCLA method to begin the process with a set of selected items, to eliminate in‐person panellist meetings in the third phase, and to enable items to be discarded between rounds.

### 2.2 Participants

The panel of experts comprised health care professionals designated by the above‐listed learned societies and the National Association of Private Midwives (ANSFL), and user representatives designated by the CIANE. In France, the role of user representatives is to defend and ensure respect for the rights and interests of users of the healthcare system. They can help improve the day‐to‐day lives of patients and their families by making their needs and problems known to decision‐makers and contributing to the production of recommendations for improving the healthcare system. The nominated panellists received an email explaining the study and containing the URL to participate in the first Delphi round. Panellists were asked to give their first name, last name and email address in the first online questionnaire. Upon completion of the first round, each respondent was given a unique identifier (6 random characters) to be entered at the beginning of each subsequent round. To ensure that a single respondent didn't complete the first round more than once, a search for duplicates was performed on first name, last name and email. For the second and third rounds, the unique identifier was used for duplicates.

### 2.3 Delphi Study

The steering committee reviewed the literature published in 2010–2022. A Pubmed query searched for the keywords “birth plan” (*n* = 2566), “birth plans” (*n* = 8979), “birth planning” (*n* = 8979) and “birth plan and satisfaction” (*n* = 103). We completed the search by reviewing the references of the selected articles, searching for reports via the websites of learned societies, the World Health Organization, and publishers of French‐language books. We finally selected 160 references and 10 websites (Supporting Information S1: File 1).

During videoconferences (19 from December 2021 through June 2023), the steering committee established an exhaustive list of components for birth plans divided into sections and subsections, with two to four formulations suggested for each. The steering committee also drafted a preamble to the birth plan intended for the pregnant woman and his/her partner. The Delphi questionnaire was then drafted and tested twice by three persons before each round.

Round 1 data were collected from November 14 through 9 December 2022, round 2 data from 3–19 January 2023, and round 3 data from February 27 through 17 March 2023. Three email reminders were sent during each round. Only panellists who completed the first round received the URL to participate in the second, and only those who completed the second round received the third‐round URL.

In each round, panellists were asked to rate individually and independently each proposed formulation of each section and subsection for its appropriateness on a 9‐point Likert scale from 1 (totally inappropriate/irrelevant) to 9 (totally appropriate/relevant), with 5 for no preference or indecisiveness. They were also asked to award their highest score to the formulation they judged most appropriate for each section and subsection and invited to comment on each formulation and its phrasing to optimize internal validity.

For round 2, panellists received descriptive statistics of the distribution of the panel's scores for each formulation rated in round 1. They were invited to re‐score the formulations not accepted in the first round on the same 9‐point Likert scale, taking the previous round answers into account. This process was repeated in round 3.

After each round, the steering committee discussed the results. Based on panellists' comments in rounds 1 or 2, the committee could recast formulations to make them more comprehensive or could add another formulation to a section or subsection. Modified and added formulations were submitted to the panellists in the next round for further evaluation.

After round 3, the Delphi results were sent for validation to the external board (28 individuals designated by the 9 learned societies listed above and the CIANE). The board reviewed the preamble and the section and subsection content during May 10–30, 2023. If two or more formulations per section or subsection were accepted during any of the rounds, the board had to choose the heading they considered most appropriate. The steering committee approved the final structure and content for birth plans during their last videoconference meeting on June 5, 2023.

### 2.4 Statistical Analysis

For statistical analysis, panellists' first names, last names and email addresses were removed from the database to make the data anonymous. The distribution of scores after each round was described by the frequencies and percentages of each score of the rating scale, the frequencies and percentages in the lowest (between 1 and 3) and highest tertiles (between 7 and 9), and the median score.

Judgement of the formulations structuring the birth plan and assessment of consensus followed the RAND/UCLA method [27]. Formulations were judged by the median score for each formulation's rating. A formulation with the panellists' median score of 7–9 was judged appropriate. A formulation with a median score of 1–3 was considered inappropriate. To evaluate the consensus between panellists (i.e., their agreement with each other), we used a continuous statistical measure of dispersion among individual scores: the Disagreement Index. We adapted the Rand Working Group definition by defining the Disagreement Index as the 10%–90% interpercentile range (IPR) divided by the IPR adjusted for symmetry (IPRAS) [27]; it is applicable to any panel size. In the RAND method, a Disagreement Index less than 1 indicates consensus or agreement between panellists (low score dispersion, with the IPRAS larger than the IPR), and a Disagreement Index exceeding 1 lack of consensus or disagreement (high score dispersion with the IPRAS smaller than the IPR) [27].

Formulations judged appropriate with agreement among the panellists were considered accepted, while those consensually rated inappropriate were considered rejected (and thus excluded in the next round). Formulations with a median score ranging between 3.5 and 6.5 or scored with disagreement between panellists were considered uncertain. Based on the round 1 findings, uncertain and/or rephrased formulations were resubmitted for further evaluation and discussion in round 2 unless another formulation for the same section or subsection had been accepted. The same process took place between rounds 2 and 3.

Statistical analyses were performed with SAS software (version 9.4, SAS Institute, Cary, NC, 2002–2012).

## 3 Results

The learned societies and the CIANE invited 46 panellists (21 health care professionals and 25 user representatives) to participate in the Delphi process. No duplicates were identified for any of the 3 rounds. The first round was completed by 42 panellists (91.3%)—19 health care professionals and 23 user representatives (95.8%) (Table 1). Round 2 was completed by 39 of the 42 experts who responded to round 1 (92.9%) (19 health care professionals and 20 user representatives), and round 3 by 36 of the 39 in round 2 (92.3%) (17 health care professionals and 19 user representatives) for an attrition rate between rounds 1 and 3 of 14.3%.

**Table 1:** Panellists' characteristics.

Characteristic	Health care professionals	Users' representatives	Total
	(*n* = 19, 45.2%)	(*n* = 23, 54.8%)	(*n* = 42)
Gender, n (%)			
Men	1 (5.3)	3 (13.0)	4 (9.5)
Women	18 (94.7)	20 (87.0)	38 (90.5)
Age (years), median (IQR)	49.0 (40.0–56.0)	41.0 (37.0–46.0)	42.5 (38.0–52.0)
Experience (years), median (IQR)	21.0 (13.0–34.0)	5.0 (1.0–11.0)	11.0 (5.0–17.0)
Health care professionals' activity, n (%)			
Midwife	13 (68.4)	—	—
Anesthesiologist	2 (10.5)	—	—
Obstetrician‐gynaecologist	2 (10.5)	—	—
Psychiatrist	2 (10.5)	—	—
Current place of health care professionals' work, n (%)			
Public health establishment	12 (63.2)	—	—
Independent practice	3 (15.8)	—	—
Territorial public service	1 (5.3)	—	—
Other	6 (31.6)	—	—

Abbreviation: IQR, Interquartile range.

Figure 1 presents the modified Delphi process used to select the birth plan content. The steering committee proposed 103 formulations for evaluation in the three Delphi rounds, divided into 8 sections (Section 1–8) and 30 subsections. The numbering and formulations of the sections and subsections are shown in Table 2.

**Table 2:** Rating scores of the formulations of sections and subsections proposed for structured content for birth plans.

Section or subsection	Formulation	First Round (*n* = 42)			Second Round^a^ (*n* = 39)			Third Round^a^ (*n* = 36)		
		Median Score^b^	DI	Accepted, Rejected, Uncertain^c^	Median Score^b^	DI	Accepted, Rejected, Uncertain^c^	Median Score^b^	DI	Accepted, Rejected, Uncertain^c^
Section 1	a. Get acquainted.	8	1.1	Uncertain	7	2.3	Uncertain			
	b. What would you like to say about yourself?	7	1.6	Uncertain	5	2.3	Uncertain			
	c. About this pregnancy.	8	1.6	Uncertain	6	2.6	Uncertain			
	d. Let's get acquainted.^d^				8	0.7	Accepted			
Subsection 1.1	a. What would you like to share about your history and your baby's?	8	1.1	Uncertain	8	1.1	Uncertain	8	2.3	Uncertain
	b. Tell us in several lines who you are.	6	2.6	Uncertain	4	2.3	Uncertain	2	1.1	Uncertain
	c. What do you want to tell us about your experience of this pregnancy?	7	2.3	Uncertain	7	2.3	Uncertain	7	2.3	Uncertain
	d. Would you like to introduce yourself in several words?	6	2.6	Uncertain	5	2.6	Uncertain	4	1.6	Uncertain
Subsection 1.2	a. Could you share with us your strengths, your resources, and the things that give you energy (sports, music, work, emotional environment, etc.)?	5	1.6	Uncertain	5	2.6	Uncertain			
	b. Would you like to share with us the strengths you can rely for this birth?	7	2.3	Uncertain	5	2.3	Uncertain			
	c. What are your resources and your strengths for this delivery?	7	1.6	Uncertain	5	2.6	Uncertain			
	d. Could you share with us what you love and value most in your life?	6	2.6	Uncertain	5	1.6	Uncertain			
	e. What are your resources and your strengths for this birth?^d^				8	0.5	Accepted			
Subsection 1.3	a. Have you prepared yourself for this birth in any specific way?	7	2.3	Uncertain	6	2.3	Uncertain	5.5	3.4	Uncertain
	b. What preparations for this birth have you made or are you planning?	7.5	1.1	Uncertain	7	2.3	Uncertain	6	3.4	Uncertain
	c. How did you or are you going to prepare for this birth?	8	1.1	Uncertain	8	2.3	Uncertain	8	2.3	Uncertain
Subsection 1.4	a. Do you have any cultural and/or symbolic information or wishes to share with us related to your delivery or the care for your baby?	6	2.6	Uncertain	5	2.6	Uncertain	5	2.3	Uncertain
	b. For this delivery, do you have any wishes related to your culture, your traditions, your beliefs, or something symbolic?	6.5	1.6	Uncertain	7	3.4	Uncertain	6.5	3.4	Uncertain
	c. What would you like to share with us of your beliefs about this pregnancy and welcoming your child?	4.5	2.6	Uncertain	3	2.3	Uncertain	3	1.6	Uncertain
	d. Do you have any symbolic cultural, spiritual, or religious concerns about this pregnancy and welcoming your child that you would like respect?	7	2.3	Uncertain	7	3.4	Uncertain	7	3.4	Uncertain
Subsection 1.5	a. We could like to take care of you and your history. What can we do in the delivery room to us help you deal with some of your personal wounds (if there are any)?	5	2.3	Uncertain	4	2.3	Uncertain			
	b. Knowing that we do not need to know all about you and your wounds, could you simply tell us what you would like us to do that will make you feel respected?	7	3.4	Uncertain	5	3.4	Uncertain			
	c. Would you like to communicate to us some aspects of your personal history — recent or long ago — that would help us to support you better?	8	1.1	Uncertain	9	0.7	Accepted			
Subsection 1.6	a. Do you have any fears or needs related to the delivery that you would like to share with the professionals who will be supporting you?	8	0.7	Accepted						
	b. Do you have anything to share with us so for that we can help you feel safe (e.g., be called by your first name, the constant presence of your partner, knock before coming in, etc.)?	8	1.6	Uncertain						
Section 2	a. Your concerns about the management of your delivery (e.g., natural labour, or after induction by medication, or a planned caesarean delivery, etc.).	6	2.3	Uncertain	5	2.6	Uncertain	3.5	1.6	Uncertain
	b. Your concerns about the management of the onset of labour (e.g., natural labour, or after induction by medication, or a planned caesarean delivery, etc.).	6	2.3	Uncertain	5	2.3	Uncertain	3	2.3	Uncertain
	c. How do you envision the start of your delivery (e.g., natural labour, after medical induction, planned caesarean delivery, etc.)?	8	3.4	Uncertain	8	3.4	Uncertain	9	0.7	Accepted
Section 3	a. Management of your labour before pushing.	5	2.3	Uncertain						
	b. Course of your labour before pushing.	6	2.3	Uncertain						
	c. Management during your contractions until it's time to push.	6	2.3	Uncertain						
	d. Your support during your contractions until it's time to push.	9	0.7	Accepted						
Subsection 3.1	a. What are your wishes for your environment during this period (e.g., light, music, bathtub, access to a shower, etc.)?	8	0.7	Accepted						
	b. What are your expectations for your environment during this period (e.g., light, music, bathtub, access to a shower, etc.)?	7	1.1	Uncertain						
	c. Do you have any needs to create yourself a cocoon, a reassuring ambiance (e.g., light, music, bathtub, access to a shower, etc.)?	7	1.6	Uncertain						
	d. Do you have any expectations or needs to feel at ease (e.g., light, music, bathtub, access to a shower, etc.)?	8	0.7	Accepted						
Subsection 3.2	a. Do you have any preferences for the management of your pain (early epidural analgesia, no epidural analgesia, other pain management methods, interventions by the adult supporting you)?	8	1.1	Uncertain	8	2.3	Uncertain	8	2.3	Uncertain
	b. Do you have any expectations for the management of your pain (early epidural analgesia, no epidural analgesia, other pain management methods, interventions by the adult supporting you)?	6.5	2.3	Uncertain	5	3.4	Uncertain	4.5	3.4	Uncertain
	c. What support do you need to deal with the intensity of your contractions (analgesia as early as possible, avoid epidural analgesia, other pain management methods, interventions by the adult supporting you)?	7.5	2.3	Uncertain	6	3.4	Uncertain	5	3.4	Uncertain
Subsection 3.3	a. What wishes do you have about your position (to walk during labour, be able to position myself as I want, use an exercise ball, suspension, etc.)?	7.5	1.6	Uncertain						
	b. Do you have any needs concerning your mobility (walking, using an exercise ball, suspension, etc.)?	6	2.3	Uncertain						
	c. How do you imagine you will be able to move (to walk during labour, be able to position yourself as you like, use an exercise ball, suspension, etc.)?	8	0.7	Accepted						
Subsection 3.4	a. What are your expectations about the other medical procedures that can be offered to you (membrane rupture, oxytocin augmentation of labour, manual vaginal examination, continuous cardiotocography, intravenous infusion, etc.)?	7	1.6	Uncertain	6	3.4	Uncertain	5	3.4	Uncertain
	b. What are your expectations about the other medical procedures that might be offered to you (membrane rupture, oxytocin augmentation of labour, manual vaginal examination, continuous cardiotocography, intravenous infusion, etc.)?	6	2.3	Uncertain	5	3.4	Uncertain	4	2.3	Uncertain
	c. Do you have any wishes about the other medical procedures that might be offered to you (membrane rupture, oxytocin augmentation of labour, manual vaginal examination, continuous cardiotocography, intravenous infusion, etc.)?	8	1.1	Uncertain	8	1.1	Uncertain	8	1.1	Uncertain
Subsection 3.5	a. Do you want to be able to drink during your delivery (water, fruit juices, energy drinks, etc.)?	8	3.4	Uncertain	8	3.4	Uncertain	8	3.4	Uncertain
	b. Your wishes about drinking (water, fruit juices, energy drinks, etc.)?	6	3.4	Uncertain	4	3.4	Uncertain	4	1.6	Uncertain
Section 4	a. Your wishes about the pushing phase?	6	1.6	Uncertain						
	b. Your expectations about the pushing phase?	5.5	2.3	Uncertain						
	c. Your wishes concerning your support during the pushing phase.	8	0.7	Accepted						
	d. Your expectations concerning your support during the pushing phase.	7	2.3	Uncertain						
Subsection 4.1	a. Do you have any wishes about the ambience and equipment in the delivery room (light, music, ability to use a mirror to see the baby's head, etc.)?	8	0.7	Accepted						
	b. Do you have any expectations about the ambience and equipment in the delivery room (light, music, ability to use a mirror to see the baby's head, etc.)?	7	1.6	Uncertain						
Subsection 4.2	a. Do you have any wishes about the place and presence (or absence) of the adult with you at the baby's emergence?	8	0.7	Accepted						
	b. What are your expectations about the place and presence (or absence) of the adult with you at the baby's emergence?	8	1.1	Uncertain						
Subsection 4.3	a. In what posture do you imagine you will give birth to your child (lying on your left side, squatting, etc.)?	7	1.6	Uncertain	5	3.4	Uncertain			
	b. Do you have a preference about your position at your child's emergence (lying on your left side, squatting, etc.)?	8	2.3	Uncertain	7	3.4	Uncertain			
	c. In what posture do you imagine yourself pushing (lying on your left side, squatting, etc.)?	7	2.3	Uncertain	5	2.3	Uncertain			
	d. In what position do you imagine you will give birth to your child (lying on your side, squatting, etc.)?^d^				9	0.7	Accepted			
Subsection 4.4	a. What are your wishes about the type of pushing?	6	2.3	Uncertain	5	3.4	Uncertain	4	3.4	Uncertain
	b. What are your expectations about the type of pushing?	5.5	2.3	Uncertain	4	2.3	Uncertain	3	2.3	Uncertain
	c. Do you want to share with the team how you have prepared yourself for the pushing phase?	8	1.1	Uncertain	8	1.6	Uncertain	8	0.7	Accepted
Subsection 4.5	a. Do you have any wishes about perineal protection: perineal massage, warm compresses, etc.?	7	1.1	Uncertain	6	3.4	Uncertain			
	b. Do you have any expectations about perineal protection: perineal massage, warm compresses, etc.?	5.5	2.3	Uncertain	5	2.3	Uncertain			
	c. Do you want to talk with professionals about perineal protection: perineal massages, warm compresses, etc.?	9	1.1	Uncertain	9	0.7	Accepted			
Subsection 4.6	a. An episiotomy (perineal incision) can be necessary. Do you have any concerns/worries about this procedure?	5	2.3	Uncertain	4	2.3	Uncertain	2.5	1.1	Uncertain
	b. If necessary and with your consent, a perineal incision (episiotomy) can be performed. Do you have any concerns about this procedure?	7.5	1.6	Uncertain	7	1.6	Uncertain	8	2.3	Uncertain
	c. An episiotomy, in some cases, can be useful for perineal protection or to accelerate the child's birth. Do you want to express any concerns or expectations?	6.5	1.6	Uncertain	7	2.3	Uncertain	7	3.4	Uncertain
Section 5	a. Whoever is at your side.	5.5	3.4	Uncertain	5	2.3	Uncertain	3	1.6	Uncertain
	b. About the person or persons with you in the delivery room.	7	1.6	Uncertain	7	3.4	Uncertain	7	3.4	Uncertain
	c. Your expectations or needs about support for you (presence of your partner or another person close to you).	7	1.6	Uncertain	7	2.3	Uncertain	8	3.4	Uncertain
Subsection 5.1	a. By whom would you wish to be supported (partner, family, friend)?	8	1.1	Uncertain						
	b. Who would you like to have at your side?	8.5	0.5	Accepted						
Subsection 5.2	a. Does the adult supporting you have expectations or needs that should be shared if delivery is vaginal?	6	2.3	Uncertain	5	2.3	Uncertain	4	1.6	Uncertain
	b. Has the adult accompanying you made any requests if delivery is vaginal?	8	1.6	Uncertain	5	2.3	Uncertain	3	2.3	Uncertain
	c. Does the adult accompanying you wish to express any requests if delivery is vaginal?^d^				9	1.6	Uncertain	9	3.4	Uncertain
Subsection 5.3	a. Does the adult supporting you have expectations or needs that should be shared if delivery is caesarean?	7	2.3	Uncertain	6	3.4	Uncertain	6	3.4	Uncertain
	b. Do you or your supporting adult want to state any requests if delivery is caesarean?	8	1.1	Uncertain	8	3.4	Uncertain	8	3.4	Uncertain
Subsection 5.4	a. What is your point of view about the presence of students?	8	3.4	Uncertain	8	3.4	Uncertain	6	3.4	Uncertain
	b. Do you have any expectations about the presence of students?	5.5	3.4	Uncertain	4	3.4	Uncertain	3	3.4	Uncertain
	c. Would you agree to the presence of students around you at delivery when appropriate?^d^							9	3.4	Uncertain
Section 6	a. Just after the birth.	9	0.3	Accepted						
	b. Immediately after the birth.	6.5	2.3	Uncertain						
Subsection 6.1	a. Do you have any wishes about when the cord will be cut and by whom?	8.5	0.7	Accepted						
	b. Do you have any expectations concerning the cutting of the cord (delayed section, cut by partner, etc.)?	6	2.3	Uncertain						
Subsection 6.2	a. Do you have any wishes about the expulsion of your placenta (active management of the third stage of labour, etc.)?	7	2.3	Uncertain	5	3.4	Uncertain	4.5	2.3	Uncertain
	b. Do you have any concerns about the expulsion of your placenta (active management of the third stage of labour, etc.)?	6	2.3	Uncertain	6	2.3	Uncertain	4.5	2.3	Uncertain
	c. Do you want to share any wishes or concerns about the expulsion of your placenta (active management of the third stage of labour, etc.)?	8	1.1	Uncertain	8	1.6	Uncertain	8.5	0.7	Accepted
Subsection 6.3	a. Do you have any wishes about cocooning your baby: skin‐to‐skin with you or your accompanying adult, no separation from the parents unless necessary, etc.?	7	2.3	Uncertain						
	b. Do you have any expectations about welcoming the baby: skin‐to‐skin with you or your supporting adult, no separation from the parents unless necessary, etc.?	8	0.7	Accepted						
Subsection 6.4	a. Do you have any wishes about the baby's care at birth (weighing, clinical examination, presence of supporting adult in the case of resuscitation, administration of vitamin K, etc.)?	8	0.7	Accepted						
	b. Do you want to talk about the newborn's care at birth (weighing, clinical examination, presence of partner in the case of resuscitation, vitamin K administration, etc.)?	9	1.1	Uncertain						
Subsection 6.5	a. What are your wishes for feeding your baby: welcome breastfeeding, early breastfeeding, milk formula, etc.?	9	0.5	Accepted						
	b. Would you like to talk to professionals about feeding your baby: welcome breastfeeding, early breastfeeding, milk formula, etc.?	8	2.3	Uncertain						
Section 7	a. Support in the days following the birth.	8.5	0.7	Accepted						
	b. Your wishes concerning your care by perinatal professionals in the days after your child's birth.	5	2.3	Uncertain						
	c. In the days after your child's birth.	7.5	2.3	Uncertain						
Subsection 7.1	a. Do you have any wishes about your support in the days after the child's birth (your partner's presence, visits, duration of hospitalization if you remain in the maternity ward)?	8	0.5	Accepted						
	b. Do you have any expectations about your support in the days after the child's birth (your partner's presence, visits, duration of hospitalization if you remain in the maternity ward)?	8	1.1	Uncertain						
Subsection 7.2	a. Do you have any wishes about the baby's care (feeding, bathing, sleeping, skin‐to‐skin, neonatal screening)?	7	1.1	Uncertain						
	b. How do you want to be supported in caring for your baby (feeding, bathing, bedding/sleeping, skin‐to‐skin, neonatal screening)?	9	0.7	Accepted						
Subsection 7.3	a. What skills do you want to develop in the immediate postpartum?	4.5	3.4	Uncertain	3	1.6	Uncertain	2	1.1	Uncertain
	b. What procedures do you want to know how to do in the days after your child is born?	9	3.4	Uncertain	9	1.6	Uncertain	9	0.7	Accepted
Subsection 7.4	a. What will you need to take care of yourself (discussions with the team, interview with a psychologist, etc.)?	8	2.3	Uncertain	7	2.3	Uncertain			
	b. What additional support would you like for yourself (discussions with the team, interview with a psychologist, etc.)?	7.5	2.3	Uncertain	8	0.7	Accepted			
Section 8	a. Besides the points raised in this document, are there any other points that you would like to raise?	8	0.7	Accepted						
	b. Do you want to attract the professionals' attention to any problems or situations not described in the other sections?	6	2.3	Uncertain						
	c. Are there any topics missing from this document that you would like to discuss?	8	1.1	Uncertain						

Abbreviation: DI, Disagreement Index.

a Formulations accepted in the first round (respectively second round) were not included in the second round (respectively third round). Formulations uncertain in the first round (respectively second round) were resubmitted in the second round (respectively third round), unless another formulation for the same section or subsection has been accepted.

^b^ Each formulation was rated on a 9‐point Likert scale from 1 (totally inappropriate/irrelevant) to 9 (totally appropriate/relevant), with 5 for indecision.

^c^ A formulation consensually judged appropriate (median score of 7–9 and DI < 1) was accepted. A formulation consensually judged inappropriate (median score of 1–3 and DI < 1) was rejected. A formulation with a median score of 3.5–6.5 or scored nonconsensually (DI > 1) was considered uncertain.

^d^ Another formulation could be added to a section or subsection considering the panellists' comments from rounds 1 or 2. Added formulations were submitted to the panellists in the next round.

The results of the three Delphi rounds are presented in Table 2. After round 1, formulations of 5 sections and 13 subsections (2 for the same subsection 3.1) were accepted. The formulations “*Your support during your contractions until it's time to push*”, “*Your wishes about the pushing phase?*”, “*Just after the birth*”, “*Support in the days following the birth*” and “*Besides the points raised in this document, are there any other points that you would like to raise?”* were accepted for Sections 3, 4, 6, 7 and 8 respectively. In Section 3, both the formulations “*What are your wishes for your environment during this period?*” and “*Do you have any expectations or needs to feel at ease?*” were accepted for Subsection 3.1. No formulation was rejected. Formulations of three sections (Sections 1, 2 and 5) and 18 subsections were considered uncertain and presented for re‐evaluation in round 2. The panellists' comments led the steering committee to add four formulations (for Section 1, subsections 1.2, 4.3, and 5.2) and slightly rephrase another.

After round 2, the formulation of Section 1 added after round 1 (“*Let's get acquainted*”) was accepted, as well as the formulations added for subsections 1.2 and 4.3. In addition, three formulations of subsections were accepted (1.5, 4.5, and 7.4). No formulation was rejected. Formulations of Sections 2 and 5 and 13 subsections were considered uncertain and presented again in round 3. Based on the panellists' comments, one formulation for subsection 5.4 was added and four were slightly rephrased.

After round 3, the formulation “*How do you envision the start of your delivery?*” of Section 2 was accepted. The formulations of the three subsections 4.4, 6.2, and 7.3 were also accepted. No formulation was rejected. Formulations of the Section 5, and 10 subsections remained uncertain.

No formulation was accepted for Section 5, and among its subsections, only one formulation was accepted for subsection 5.1. The steering committee then decided to retain the accepted formulation of subsection 5.1 as the formulation of Section 5.

The third‐round Delphi results were transferred to the external board for validation (21 of 28 responses were submitted, for a response rate of 75%: 18 professionals and 3 user representatives). The preamble to the birth plan and the content presented in the sections and subsections were reviewed and approved. The panel had accepted two formulations for subsection 3.1; the external board judged the formulation “*Do you have any expectations or needs to feel at ease?*” most appropriate. During their last videoconference meeting, the steering committee approved the preamble to the birth plan, which was designed to explain the benefits of a birth plan to the woman and his/her partner (Supporting Information S1: File 2). The steering committee also approved the final components of the birth plan structure into 8 sections and 19 subsections. Finally, the 8 sections were: “*Let's get acquainted*”, “*How do you envision the start of your delivery*”, “*Your support during your contractions until it's time to push*”, “*Your wishes concerning your support during the pushing phase*”, “*Who would you like to have at your side?*” “*Just after the birth*”, “*Support in the days following the birth*”, and “*Besides the points raised in this document, are there any other points that you would like to raise?*”. Table 3 details the 8 sections and 19 subsections. The final results were communicated to users and perinatal caregivers through a French practice advisery.

**Table 3:** Final content of the structured birth plan obtained with a Delphi process.

**1. Let's get acquainted.**
What are your resources and your strengths for this birth?
Would you like to communicate to us some aspects of your personal history — recent or long ago — that would help us to support you better?
Do you have any fears or needs related to the delivery that you would like to share with the professionals who will be supporting you?
**2. How do you envision the start of your delivery (e.g., natural labour, after medical induction, planned caesarean delivery, etc.)?**
**3. Your support during your contractions until it's time to push.**
Do you have any expectations or needs to feel at ease (e.g., light, music, bathtub, access to a shower, etc.)?
How do you imagine you will be able to move (to walk during labour, be able to position yourself as you like, use an exercise ball, suspension, etc.)?
**4. Your wishes concerning your support during the pushing phase.**
Do you have any wishes about the ambience and equipment in the delivery room (light, music, ability to use a mirror to see the baby's head, etc.)?
Do you have any wishes about the place and presence (or absence) of the adult with you at the baby's emergence?
In what position do you imagine you will give birth to your child (lying on your side, squatting, etc.)?
Do you want to share with the team how you have prepared yourself for the pushing phase?
Do you want to talk with professionals about perineal protection: perineal massages, warm compresses, etc.?
**5. Who would you like to have at your side?**
**6. Just after the birth.**
Do you have any wishes about when the cord will be cut and by whom?
Do you want to share any wishes or concerns about the expulsion of your placenta (active management of the third stage of labour, etc.)?
Do you have any expectations about welcoming the baby: skin‐to‐skin with you or your supporting adult, no separation from the parents unless necessary, etc.?
Do you have any wishes about the baby's care at birth (weighing, clinical examination, presence of supporting adult in the case of resuscitation, administration of vitamin K, etc.)?
What are your wishes for feeding your baby: welcome breastfeeding, early breastfeeding, milk formula, etc.?
**7. Support in the days following the birth.**
Do you have any wishes about your support in the days after the child's birth (your partner's presence, visits, duration of hospitalization if you remain in the maternity ward)?
How do you want to be supported in caring for your baby (feeding, bathing, bedding/sleeping, skin‐to‐skin, neonatal screening)?
What procedures do you want to know how to do in the days after your child is born?
What additional support would you like for yourself (discussions with the team, interview with a psychologist, etc.)?
**8. Besides the points raised in this document, are there any other points that you would like to raise?**

## 4 Discussion

The use of a modified Delphi method produced structured content for birth plans in 8 sections and 19 subsections. The content was based simultaneously on the literature and on the judgements of a panel of health care professionals and user representatives.

Although birth plans were introduced in the 1980s, few countries have reached a consensus on their structure or content [24]. It is also difficult to assess the impact of birth plans on childbearing women's experiences or on maternal and neonatal and infant outcomes, especially given the heterogeneity of the non‐standardized birth plans among the publications included in the systematic reviews and meta‐analyses [22, 23].

The literature describes the content of birth plans by various combinations of terms: preferences, expectations, wishes, requests, desires, views, demands, and values [23]. According to Bell et al., the most common term is preferences [23]. These variations may be linked to the cultural contexts of the published studies. In our Delphi process, finally, the more frequently used terms (13 times) were: “*would you like*” or “*do you have any wishes*”. We used an interrogative form for 3 of the 8 sections and for all 19 subsections.

Our structured birth plan includes 8 sections and 19 subsections; women (or couples) could choose not to answer each proposal. Scotland's birth plan is divided into 11 sections (with 5 interrogative propositions) [25]. The NHS birth plan template has 17 non‐interrogative sections, each including multiple suggestions to be checked and the ability to write [26]. Scotland's template includes an introduction, as do we; our preamble explains to users what a birth plan is, its modifiability throughout pregnancy and childbirth, and the multiplicity of user‐preferred formats accepted (Supporting Information S1: File 2). During its first meetings, the steering committee decided to include, as the Scottish version does, questions about the postpartum period.

Because the steering committee included perinatal psychologists and psychiatrists, the first draft questionnaire included a variety of symbolic, spiritual, religious or cultural formulations. These proposals were rejected with one commenter stating that these questions were too intrusive.

The expert panel did not accept questions about the pain during childbirth. The most likely explanation is that French pregnant women know that they can ask for analgesia during labour. Most do for vaginal deliveries (82.7% of epidural analgesia), and only 3.5% were not at all satisfied with the pain relief methods used during childbirth [17]. Pain management is addressed during the mandatory prenatal consultations; birth and parenthood preparation sessions, and at the last trimester of pregnancy during the mandatory pre‐delivery anaesthesiology consultation. In any case, the very open final section can be used to address pain relief.

The question about episiotomies was rejected, both because French pregnant women are already known to prefer avoiding episiotomy and because professionals have regularly updated guidelines concerning perineal protection during childbirth [31, 32]. Episiotomy rates fell from 20.1% in 2016% to 8.3% in 2021 [17].

### 4.1 Clinical Implications

Some challenges remain in improving the adherence of pregnant women and perinatal care providers to creating and following birth plans. Reaching a national consensus with a large number of relevant learned societies and users is an essential first step before implementing actions to promote the drafting of birth plans.

Perinatal care providers have had a poor perception of birth plans because of the inflexibility of some of their elements and some requests believed to be inappropriate or not evidence‐based [33]. The joint construction of a structured birth plan by professionals and users may improve these providers' acceptance of birth plans and thus improve the communication and shared decision‐making between these two groups. Birth plans are a tool that supports perinatal professionals in taking the time to listen to pregnant women about what matters to them at birth and postpartum *—* surely the most effective way to avoid subsequent conflict. Discussing perinatal and neonatal outcomes in relation to what had been written in the birth plan is also a useful practice that can reduce posttraumatic stress or postnatal depression and improve pregnant women's satisfaction.

### 4.2 Strengths and Limitations

One of the strengths of this work is the use of the Delphi method, a group facilitation technique designed to reach consensus on expert opinions through a series of structured questionnaires, commonly referred to as rounds. This method has the advantage of enabling the participation of a large number of experts, often remotely, through online questionnaires. It also allows experts to express their opinions anonymously and independently, thus preventing any dominance of the group by the views of certain individuals. The content of the birth plan was developed simultaneously based on current scientific literature and designed to ensure its applicability in practice. This multistep process took into account the feedback from the panellists, who were health care professionals and user representatives, to make the components of the birth plan as understandable as possible and to ensure its acceptability in clinical practice. The number of panellists in the Delphi process is another strength of our study [34, 35, 36]. To our knowledge, there are currently no clear guidelines for the sample size of Delphi studies. A minimum number of experts between 7 and 15 has been suggested [27, 35]. The larger panel in our study should have optimized the reliability of the final content of the proposed birth plan [34, 37]. Moreover, only six panellists from the first round did not respond to the third round and the follow‐up response rate (85.7%) exceeded the 70% suggested [38]. The majority of the panellists were midwives which is a strength. Indeed, in France, more than half of all births, in public and private maternity hospitals and in birth centres, are carried out by a midwife (57.1% in 2021), and midwives account for 88.6% of all spontaneous vaginal deliveries alone [17]. The steering committee directing this study was chaired by a national users' association, and users were represented in all three working groups. Ten French perinatal societies supported and participated in this work. We adhered to the rigorous process of a Delphi study. To our knowledge, only Scotland has a national structure and content for birth plans, but they did not explain the method to obtain a consensus [24, 25].

Our study has some limitations. It is not an international Delphi process, but it was based on an English and French literature review and accordingly enables fairly good external validity for most countries, French‐speaking or not. We intentionally limited the number of rounds to three to avoid nonresponse as much as possible. We did not compare the results between health care professionals and user representatives, as this was not our objective, which was to achieve a national consensus among perinatal professionals and user representatives. However, from a sociological perspective, such an analysis would be interesting. Finally, our work focused only on the structure and content of birth plans, without a parallel nationwide project to develop guidelines on how, when, and with whom to write birth plans.

## 5 Conclusion

A national Delphi process conducted in collaboration with users and 9 perinatal societies, included three rounds of scoring, enabling a consensus on the structure and content of 8 sections and 19 subsections for birth plans. Challenges related to the use of this structured birth plan by perinatal caregivers and pregnant women (couples) remain. A multicenter randomized controlled clinical trial with as its intervention a national standardized birth plan should then assess the effects of birth plans on the efficiency of prenatal visits, pregnant women's experiences of childbirth and on obstetric and neonatal outcomes.

## Author Contributions

**Françoise Vendittelli:** conceptualization; formal analysis, funding acquisition, investigation, methodology, project administration, supervision, validation, visualization, writing–original draft, writing–review and editing. **Lucie Adalid:** investigation, project administration, validation, visualization, writing–review and editing. **Violaine Peyronnet:** investigation, project administration, validation, visualization, writing–review and editing. **Sophie Guillaume:** investigation, project administration, validation, visualization, writing–review and editing. **Nathalie Piquée:** investigation, project administration, validation, visualization, writing‐review and editing. **Aurore Viard‐Cretat:** investigation, project administration, validation, visualization, writing–review and editing. **Catherine Crenn‐Hébert:** investigation, project administration, validation, visualization, writing–review and editing. **Olivier Rivière:** data curation, investigation, project administration, resources, software, validation, visualization, writing‐review and editing. **Candy Guiguet‐Auclair:** data curation, formal analysis, investigation, methodology, project administration, resources, software, validation, visualization, writing‐original draft, writing–review and editing. **Study group:** project administration, validation, writing‐review and editing.

## Study Group

Lucie Adalid, Ms. (CIANE, France); Claire Audouin, Ms. (SFMP, France); Violaine Peyronnet, MD (Hôpital Louis Mourier, HPHP, CNGOF, France); Catherine Crenn‐Hébert, MD (Hôpital Louis Mourier, HPHP, Audipog, France); Candy Guiguet‐Auclair, PhD (Centre Hospitalier Universitaire de Clermont‐Ferrand, France); Laurent Gerbaud, MD, PhD (Centre Hospitalier Universitaire de Clermont‐Ferrand, France); Sophie Guillaume, Ms. (APHP, CNSF, France); Cyril Huissoud, MD, PhD (Centre Hospitalier Universitaire de Lyon, CNGOF, France); Françoise Molenat (Montpellier, France); Estelle Morau Ms. (CARO, France); Sylvie Le Roux, Ms. (ANSFC, France); Nathalie Piquée, Ms. (CNSF, France), Olivier Rivière (Audipog, France); Françoise Vendittelli, MD, PhD (Centre Hospitalier Universitaire de Clermont‐Ferrand, Audipog, France); Aurore Viard‐Cretat, PhD (CIANE, France).

## Ethics Statement

Local Ethics Committee (IRB00013412, “CHU de Clermont Ferrand IRB #1,” IRB number 2022‐CF066) approved the study, which adhered to French individual data protection regulations. All panellists consented to participate in the Delphi survey.

## Conflicts of Interest

The authors declare no conflicts of interest.

## Supporting information

Supporting information.

## Data Availability

The data that support the findings of this study are available from the corresponding author upon reasonable request.
