# Supplementary material for: Consensus on the Structure and Content of Birth Plans: A Modified Delphi Study
Source: Health Expect. 2024 Dec 18;27(6):e70124. doi: 10.1111/hex.70124 (PMC11655921; doi:10.1111/hex.70124)
Supplement: Supplementary file 1 — Supporting information. [file HEX-27-e70124-s001.docx]

**Supplementary Files**

**Supplementary File 1**: References and websites obtained through the steering committee’s literature research.

**Supplementary file 2**: Preamble to the structured birth plan intended for the woman and her partner.

**Supplementary File 1**: References and websites obtained through the steering committee’s literature research.

1. Abd El Aliem RS, Eman AM, El Ahmady Sarhan A. Effect of implementing birth plan on women childbirth outcoms and empowerment. Am J Nurs Sc 2020 ;9:160-70.
2. ACOG Committee Opinion No. 766: Approaches to limit intervention during labor and birth. Obstet Gynecol 2019;133:e164-e173.
3. Afshar Y, Mei J, Gregory K. Birth plans: Birth preferences or labor manifesto. Gynecology & Obstretrics Case report 2016;2:1-4.
4. Afshar Y, Wang ET, Mei J, Esakoff TF, Pisarska MD, Gregory KD. Childbirth education class and birth plans are associated with a vaginal delivery. Birth 2017;44:29-34.
5. Afshar Y, Mei JY, Gregory KD, Kilpatrick SJ, Esakoff TF. Birth plans-impact on mode of delivery, obstetrical interventions, and birth experience satisfaction: A prospective cohort study. Birth 2018;45:43-9.
6. Afshar Y, Mei J, Fahey J, Gregory KD. Birth plans and childbirth education: What are provider attitudes, beliefs, and practices? J Perinat Educ 2019;28:10-8.
7. Ahmadpour P, Mosavi S, Mohammad-Alizadeh-Charandabi S, Jahanfar S, Mirghafourvand M. Evaluation of the birth plan implementation: A parallel convergent mixed study. Reprod Health 2020;17:138.
8. Alba-Rodríguez R, Coronado-Carvajal MP, Hidalgo-Lopezosa P. The birth plan experience - A pilot qualitative study in southern spain. Healthcare 2022;10:95.
9. Alvarez L, Cayol V. Psychologie et psychiatrie de la grossesse. De la femme à la mère. Eds *Odile Jacob*. Paris, France; 2015.
10. Anderson CM, Monardo R, Soon R, Lum J, TschannM, Kaneshiro B. Patient communication, satisfaction, and trust before and after use of a standardized birth plan. Hawaii J Med Public Health 2017;76:305-9.
11. Aragon M, Chhoa E, Dayan R, Kluftinger A, Lohn Z, Buhler K. Perspectives of expectant women and health care providers on birth plans. J Obstet Gynaecol Can 2013;35:979-85.
12. Armstrong N, Kenyon S. When choice becomes limited: Women’s experiences of delay in labour. Health 2017;21: 223-38.
13. Bailey JM, Crane P, Nugent CE. [Childbirth education and birth plans.](https://pubmed.ncbi.nlm.nih.gov/18760232/) Obstet Gynecol Clin North Am 2008;35:497-509.
14. Barandon S, Balès M, Melchior M, et al. Entretien prénatal précoce et séances de préparation à la naissance et à la parentalité : caractéristiques psychosociales et obstétricales associés chez les femmes de la cohorte Elfe. J Gynecol Obstet Biol Reprod (Paris) 2016;45:599-607.
15. Barraux L, Storme B, Leymarie MC. Une enquête de satisfaction auprès des patientes ayant rédigé un projet de naissance. Vocation Sages-Femmes 2015;117:18-21.
16. Barimani M, Forslund Frykedal K, Rosander M, Berlin A. Childbirth and parenting preparation in antenatal classes. Midwifery 2018;57:1-7.
17. Bayle B. L’enfant à naître. Identité conceptionnelle et gestation psychique. Eds *Erès*. Toulouse, France; 2005.
18. Bayle B. Maternité et traumatismes sexuels de l’enfance. Une clinique de l’interface soma-psyché. Eds *L’Harmattan*. Paris, France; 2006.
19. Bayle B. Ma mère est schizophrène. Schizophrénie et parentalité, collection « La vie de l’enfant ». Eds *Erès*. Toulouse, France; 2008.
20. Bayle B. Perdre son jumeau à l’aube de la vie, collection « La vie de l’enfant ». Eds *Erès*. Toulouse, France; 2013.
21. Bayle B. Le déni de grossesse, un trouble de la gestation psychique, collection « La vie de l’enfant ». Eds *Erès*. Toulouse, France; 2016.
22. Bayle B. Aide-Mémoire de Psychiatrie et psychopathologie périnatales. Eds *Dunod*. Paris, France; 2017.
23. Bayle B. Traumatismes psychiques à l’aube de la vie, collection La Vie de l’enfant. Eds *Erès*. Toulouse, France; 2021.
24. Bailey JM, Crane P, Nugent CE. Childbirth education and birth plans. Obstet Gynecol Clin North Am 2008;35:497-509.
25. Bell CH, Muggleton S, Davis DL. Birth Plans: A systematic, integrative review into their purpose, process, and impact. Midwifery 2022;111:103388.
26. Berg M, Lundgren I, Lindmark G. Childbirth experience in women at high risk: Is it improved by use of a birth plan? J Perinat Educ 2003;12:1-15.
27. Bringedal H, Aune I. Able to choose? Women’s thoughts and experiences regarding informed choices during birth. Midwifery 2019;77:123-9.
28. Brooks H, Sullivan WJ. The importance of patient autonomy at birth. Int J Obstet Anesth 2002;11:196-203.
29. Brown SJ, Lumley J. Communication and decision-making in labour: Do birth plans make a difference? Health Expect 1998;1:106-16.
30. Brown S, Lumley J. Satisfaction with care in labor and birth: A survey of 790 Australian women. Birth 1994;21:4-13.
31. Bydlowski M. Devenir mère. Eds *Odile Jacob*. Paris, France; 2020.
32. Carquillat P, Boulvain M, Guittier MJ. How does delivery method influence factors that contribute to women’s childbirth experiences? Midwifery 2016;43:21-8.
33. Carty EM, Tier DT. Birth planning: A reality based script for confidence building. J Nurse Midwifery 1989;34:111-3.
34. Chalmers B, Mangiaterra V, Porter R. WHO principles of perinatal care: the essential antenatal, perinatal, and postpartum care course. Birth 2001;28:202-7.
35. Collet M. Satisfaction des usagères des maternités à l’égard du suivi de grossesse et du déroulement de l’accouchement. Drees, Etudes et résultats 2008;660:1-6.
36. Cook K, Loomis C. The impact of choice and control on women’s childbirth experiences. J Perinat Educ 2012;21:158-68.
37. Cortezzo DE, Bowers K, Cameron Meyer M. Birth planning in uncertain or life-limiting detal diagnoses: Perspectives of physicians and parents. J Palliat Med 2019;22:1337-45.
38. Crossland N, Kingdon C, Balaam MC, Betran AP, Downe S. Women’s, partners’ and healthcare providers’ views and experiences of assisted vaginal birth: A systematic mixed methods review. Reprod Health 2020;17:83.
39. Davis B, Clark S, McDonald K, young T, Swain L. The impact of a birth plan on maternal and neonatal delivery outcomes. Am J Obstet Gynecol 2019;22:309.
40. Davis-Floyd R. The technocratic, humanistic, and holistic paradigms of childbirth. Int J Gynaecol Obstet 2001:75,S5–S23.
41. DeBaets AM. From birth plan to birth partnership: Enhancing communication in childbirth. Am J Obstet Gynecol 2017;216:31.e1–31.e4.
42. Deering SH, Zaret J, McGaha K, Satin AJ. Patients presenting with birth plans: A case-control study of delivery outcomes. J Reprod Med 2007;10:884–7.
43. Deering MA, Heller J, McGaha K, Heaton J, Satin AJ. Patients presenting with birth plans in a military tertiary care hospital: A descriptive study of plans and outcomes. Mil Med 2006;171:778-80.
44. Divall B, Spiby H, Roberts J, Walsh D. Birth plans: A narrative review of the literature. Int J of Childbirth 2016;6:157-72 .
45. Divall B, Spiby H, Nolan M, Slade P. Plans, preferences or going with the flow: An online exploration of women’s views and experiences of birth plans. Midwifery 2017;54:29-34.
46. Delassus JM. L’aide-mémoire de la maternologie. Eds Dunod. Paris, France; 2010.
47. Delassus JM. Le sens de la maternité. Eds *Dunod*. Paris, France; 2011.
48. Delassus JM. Penser la naissance. Eds *Dunod*. Paris, France; 2011.
49. Divall B, Spiby H, Roberts J, Walsh D. Birth plans: A narrative review of the literature. Int J Child 2016;6:157-72.
50. Doherty M. Birth plan decision-making patterns of interaction. Int J Childbirth Educ 2003;18:27-33.
51. Dugnat M, Glangeaud-Freudenthal N, Isserlis C. Guide pour la pratique de l’entretien prénatal précoce. Eds *Erès*, Toulouse, Frabce; 2008.
52. Dugnat M. Devenir père, devenir mère. Naissance et parentalité. Eds *Erès*. Toulouse, France; 2012.
53. Ekeocha CEO, Jackson P. The « birth plan » experience. BJOG 1985;92:97-101.
54. Farahat AH, Mohamed HES, Elkader SA, El-Nemer A. Effect of implementing a birth plan on womens’ childbirth experiences and maternal & neonatal outcomes. J of Educ and Pract 2015;6:24-31.
55. Ferry N. Devenir mère, une formidable rencontre. Spirale 2008;47:157-65.
56. Gamelin-Lavois, S. Préparer son accouchement. Faire un projet de naissance. Eds *Jouvence*. Saint-Julien-en-Genevois, Suisse; 2006.
57. Gamelin-Lavois S. La naissance autrement. Réaliser son projet de naissance. Eds *Jouvence*. Saint-Julien-en-Genevois, Suisse; 2014.
58. Ghanbari-Homayi S, Hasani S, Meedya S, Asghari Jafarabadi M, Mirghafourvand M. Nonpharmacological approaches to improve women’s childbirth experiences: A systematic review and meta-Analysis. J Matern Fetal Neonatal Med 2021;34:479-91.
59. Gibbins J, Thomson AM. Women’s expectations and experiences of childbirth. Midwifery 2001;17:302-13.
60. Glangeaud-Freudenthal NMC, Gressier F. Accueillir les pères en périnatalité. La vie de l’enfant. Eds *Erès*. Toulouse, France; 2017.
61. Gottfredsdottir H, Steingrimsdottir T, Bjornsdottir A, Guethmundsdottir EY, Kristjansdottir H. Content of antenatal care: Does it prepare women for birth? Midwifery 2016;39: 71-7.
62. Grant R, Sueda A, Kaneshiro B. Expert opinion vs. patient perception of obstetrical outcomes in laboring women with birth plans. J reprod Med 2010;55:31-5.
63. Gultie T, Tanto Z, Estifanos W, Boti N, de Courten B. Husbands' participation in birth preparedness and complication readiness plan in Kucha district, Gamo Zone, Southern Ethiopia. PLoS One. 2021;16:e0261936.
64. Hadar E, Raban O, Gal B, Yogev Y, Melamed N. Obstetrical outcome in women with self-prepared birth plan. J Matern Fetal Neonatal Med 2012;25: 2055-57.
65. Hadian T. Effect of birth plans on childbirth experience: A systematic review. Int J Nurs Pract. 2019;25:e12722.
66. Hands KK, Clements-Hickman A, Davies CC, Brockopp D. The effect of hospital-based childbirth classes on women’s birth preferences and fear of childbirth: A pre- and post-class survey. J Perinat Educ 2020;29: 134-42.
67. Haute Autorité de Santé (2005). Préparation à la naissance et à la parentalité (PNP). Available at: <https://www.has-sante.fr/upload/docs/application/pdf/preparation_naissance_recos.pdf>. Accessed August 17, 2023.
68. Haute Autorité de Santé (2014). Qualité et sécurité des soins dans le secteur de naissance. Guide méthodologique. Available at: <https://gynerisq.fr/wp-content/uploads/2017/11/2014_HAS-securite-secteur-naissance.pdf>. Accessed August 17, 2023.
69. Havasi K. Les pères : de la préparation à la naissance et à la parentalité (PNP) au retour à domicile. Rev Med Perinat 2017;9:230-4.
70. Henriksen L, Grimsrud E, Schei B, Lukasse M, Bidens Study Group. Factors related to a negative birth experience - A mixed methods study. Midwifery 2017;51:33-9.
71. Hidalgo-Lopezosa P, Cubero-Luna AM, Jimenez-Ruz A, Hidalgo-Maestre M, Rodriguez- Borrego MA, Lopez-Soto PJ. Association between birth plan use and maternal and neonatal outcomes in Southern Spain: A case-control study. Int J Environ Res Public Health 2021;18:456.
72. Hidalgo-Lopezosa P, Hidalgo-Maestre M, Rodriguez-Borrego MA. Birth plan compliance and its relation to maternal and neonatal outcomes. Rev Lat Am Enfermagem 2017;25: e2953.
73. Hidalgo-Lopezosa P, Rodriguez-Borrego MA, Munoz-Villanueva MC. Are birth plans associated with improved maternal or neonatal outcomes? MCN Am J Matern Child Nurs 2013;38: 150-6.
74. Hollander MH, van Hastenberg E, van Dillen J, van Pampus MG, de Miranda E, Stramrood CAI. Preventing traumatic childbirth experiences: 2192 women’s perceptions and views. Arch Womens Ment Health 2017;20: 515-23.
75. Inch S. Birth plans and protocols. J R Soc Med 1988;81:120-2.
76. Instruction N° SGMCAS/2021/74 du 1er avril 2021 relative aux engagements du Gouvernement pour l’année 2021 autour de la politique des 1000 premiers jours de l’enfant et de ses modalités de déclinaison territoriale, ainsi qu’aux leviers supplémentaires mis à disposition des acteurs locaux. Available at: <https://sante.gouv.fr/IMG/pdf/1000premiersjours-instruction-01.04.21.pdf>. Accessed August 17, 2023.
77. Isserlis C, Sutter-Dallay AL, Dugnat M, Glangeaud-Freudenthal N. Guide pour la pratique de l’entretien prénatal précoce et l’accompagnement psychique des femmes devenant mères. Eds *Erès*. Toulouse, France; 2020.
78. Jackson P. The Huddersfield birth plan. Matern Child Health 1986;11:14-7.
79. Jenkinson B, Kruske S, Kildea S. Refusal of recommended maternity care: Time to make a pact with women? Women Birth 2018;31:433-41.
80. Jenkinson B, Kruske S, Stapleton H, Beckmann M, Reynolds M, Kildea S. Women’s, midwives’ and obstetricians’ experiences of a structured process to document refusal of recommended maternity care. Women Birth 2016;29:531-41.
81. Jolles MW, de Vries M, Hollander MH, van Dillen J. Prevalence, characteristics, and satisfaction of women with a birth plan in The Netherlands. Birth 2019;46:686-92.
82. Jones MH, Barik S, Mangune HH, Jones P, Gregory SJ, Spring JE. Do birth plans adversely affect the outcome of labour? Br J Midwifery 1998;6:38-41.
83. Kaneshiro B, Grant R, Sueda A. Expert opinion vs. patient perception of obstetrical outcomes in laboring women with birth plans. J Reprod Med 2010;55:31-5.
84. Kaufman T. Evolution of the birth plan. J Perinat Educ 2007;16:47–52.
85. Kitzinger S. Sheila Kitzinger’s letter from England: Birth plans. Birth 1992;19:36-7.
86. Kitzinger S. Birth plans: How are they being used. B J Midwifery 1999;7:300-3.
87. Kitzinger S. The politics of birth. Eds *Elsevier*. Edinbourg, Scotland; 2005.
88. Knupp Medeiros RM, Figueiredo G, de Paula Correa AC, Barbieri M. Repercussions of using the birth plan in the parturition process. Rev Gaucha Enferm 2019;40:e20180233.
89. Kotaska A. Informed consent and refusal in obstetrics: A practical ethical guide. Birth 2017;44:195-9.
90. Kruske S, Young K, Jenkinson B, Catchlove A. Maternity care providers’ perceptions of women’s autonomy and the law. BMC Pregnancy Childbirth 2013;13:84.
91. Kuo SC, Lin KC, Hsu CH, et al. Evaluation of the effects of a birth plan on Taiwanese women's childbirth experiences, control and expectations fulfilment: A randomised controlled trial. Int J Nurs Stud 2010;47:806-14.
92. Labalestra M, Culot S, Gauge J. Impliquer, informer et soutenir les pères durant la période périnatale. Périnatalité. Perinat 2021;13:46-53.
93. Lagan B, Sinclair M, KernohanWG. Pregnant women’s use of the internet: A review of published and unpublished evidence. Evidence Based Midwifery 2006;4:17-23.
94. Legros JP. La préparation des futurs pères. Vers la reconnaissance d’une langue paternelle. Spirale 2008;47:91-5.
95. Lejoyeux M. Les 4 temps de la renaissance. Le stress post-traumatique n’est pas une fatalité. Eds *Jean-Claude Lattès*. Paris, France; 2020.
96. Levett K, Dahlen HG. Perspective: Childbirth education in Australia: Have we lost our way? Women Birth 2019;32:291-3.
97. Lewis L, Hauck YL, Ritchie S, Barnett L, Nunan H, Rivers C. Australian women’s perception of their preparation for and actual experience of a recent scheduled caesarean birth. Midwifery 2014;30:e131–e136.
98. López-Gimeno E, Falguera-Puig G, Vicente-Hernández MM, Angelet M, Garreta GV, Seguranyes G. Birth plan presentation to hospitals and its relation to obstetric outcomes and selected pain relief methods during childbirth. BMC Pregnancy Childbirth 2021;21:274.
99. Lopez-Toribio M, Bravo P, Llupia A. Exploring women’s experiences of participation in shared decision-making during childbirth: A qualitative study at a reference hospital in Spain. BMC Pregnancy Childbirth 2021;21:631.
100. Lothian J. Birth plans: The good, the bad, and the future. J Obstet Gynecol Neonatal Nurs 2006;35:295-303.
101. Love C, Pace N. Consent. Anaesthesia & Intensive Care Medicine 2018;19:263-6.
102. Lundgren I, Berg M, Lindmark G. Is the childbirth experience improved by a birth plan? J Midwifery Womens Health 2003;48:322-8.
103. Magoma M, Requejo J, Campbell O, Cousens S, Merialdi M, Filipp V. The effectiveness of birth plans in the increasing use of skilled care at delivery in rural Tanzania. Trop Med Int Health J 2013;18:435-43.
104. Malacrida C, Boulton T. The best laid plans? Women’s choices, expectations and experiences in childbirth. Health 2014;18:41-59.
105. McEwan E, Tier T. Birth planning. A reality-based script for building confidence. J Nurse Midwifery 1989;34:111-4.
106. McKenzie-McHarg K, Ayers S, Ford E, et al. Post-traumatic stress disorder following childbirth: An update of current issues and recommendations for future research. J Reprod Infant Psychol2015;33:219–37.
107. Medeiros RMK, Figueiredo G, Correa ACP, Barbieri M. Repercussions of using the birth plan in the parturition process. Rev Gaucha Enferm 2019;40:e20180233.
108. Mei JY, Afshar Y, Gregory KD, Kilpatrick SJ, Esakoff TF. Birth plans: What matters for birth experience satisfaction. Birth 2016;43:144-50.
109. Ministère des Solidarité et de la santé (2020). Rapport de la commission des 1000 premiers jours. Les 1000 premiers jours. Là où tout commence. Available at: <https://sante.gouv.fr/IMG/pdf/rapport-1000-premiers-jours.pdf>. Accessed August 17, 2023.
110. Ministère du travail, de l’emploi, et de la santé (2011). Suivi et accompagnement des grossesses physiologiques par les maternités. Fondamentaux. Available at: <https://sante.gouv.fr/IMG/pdf/grossesses_physiologiques.pdf>. Accessed August 17, 2023.
111. Mirghafourvand M, Charandabi SMA, Ghanbari-Homayi S, Jahangiry L, Nahaee J, Hadian T. Effect of birth plans on childbirth experience: a systematic review. Int J Nurs Pract 2019;25:e12722.
112. Missonnier S, Golse B, Soulé M. La grossesse, l'enfant virtuel et la parentalité. Eds *Presses Universitaires de France*. Paris, France; 2004.
113. Missonnier S. Devenir parent, naître humain. Eds *Presses Universitaires de France*. Paris, France; 2009.
114. Missonnier S. Grossesse, stress et psychanalyse. Un débat primordial. La psychiatrie de l'enfant 2013;56:37-66.
115. Molénat F. Prévention précoce : petit traité pour construire des liens humains. Eds *Érès*. Toulouse, France; 2009.
116. Molénat F, Toubin RM, Panagiotou D. Grossesse et prévention. Contraste 2017;46:269-302.
117. Molénat F, Morard-Dubey A, Roegiers L. Le sentiment de continuité en périnatalité. Concepts et outils : une élaboration progressive. Perinat 2019 ;11:149-60.
118. Moore M, Hopper U. Do birth plans empower women? Evaluation of a hospital birth plan. Birth.1995;22:29-36.
119. Morisseau L. Lorsque la parentalité paraît. Eds *Presses Universitaires de France*. Paris, France; 2009.
120. Morton CH, Simkin P. Can respectful maternity care save and improve lives? Birth 2019;46:391-5.
121. Ndeto J, Barase S, Murigi M, Keraka M, Osero J. Uilization of individual birth plan during pregnancy and its determinants in Makueni County. Int J Comm Med Public Health 2018;5:30-7.
122. Neyrand G. Evolution de la parentalité et rapport à l’enfant. Enjeux théoriques et débats sociaux. Perinat 2022;14:95-103.
123. Ngai F, Chan S. The effect of a childbirth psycho-education program on maternal role competence and perinatal depression: A quasi-experiment. Int J Nurs Stud 2009;46:1298-306.
124. Nilsson L, Thorsell T, Hertfelt Wahn E, Ekstrom A. Factors influencing positive birth experiences of first-time mothers. Nurs Res Pract 2013:349124.
125. O’Deyé C. Accompagner la parentalité en exil. Analyse et guide pratique à l’usage des intervenants. Eds *Presses de l’EHESP*. Rennes, France; 2021.
126. Owens KH. Confronting rhetorical disability: A critical analysis of women’s birth plans. Written Communication 2009;26:247-72.
127. Patterson J, Hollins Martin C, Karatzias T. PTSD post-childbirth: A systematic review of women’s and midwives’ subjective experiences of care provider interaction. J Reprod Infant Psychol 2019;37:56-83.
128. Peart K. Birth planning – Is it beneficial to pregnant women? Australian Midwifery 2004;17:27-9.
129. Pennell A, Salo-Coombs V, Herring A, Spielman F, Fecho K. Anesthesia and analgesia-related preferences and outcomes of women who have birth plans. J Midwifery Womens Health 2011;56:376-81.
130. Perelman O, Riazuelo H, Carvalho E, Missonnier S. Le dessin, la grossesse et l’enfant à naître. La psychiatrie de l'enfant 2021;64:5-56.
131. Perelman O, Missonnier S, Guéguen C. Identité(s) paternelle, parentale et conjugale : devenir père d’un enfant. Cliniques Méditerranéennes 2020;101:193-205.
132. Preis H, Lobel M, Benyamini Y. Between expectancy and experience: Testing a model of childbirth satisfaction. Psychology of Women Quarterly 2018;43:105–17.
133. Razurel C, Antonietti JP, Rulfi F, Pasquier N, Domingues-Montanari S, Darwiche J. The impact of pre- and post-natal psycho-educational intervention on the construction of parenthood. Arch Womens Ment Health 2017;20:469-72.
134. Reed R, Sharman R, Inglis C. Women’s descriptions of childbirth trauma relating to care provider actions and interactions. BMC Pregnancy Childbirth 2017;17:21.
135. Regan M, McElroy KG, Moore K. Choice? Factors that influence women’s decision making for childbirth’. J Perinat Educ 2013;22:171-80.
136. Rodríguez-Almagro J, Hernández-Martínez A, Rodríguez-Almagro D, Quirós-García JM, Martínez-Galiano JM, Gómez-Salgado J. Women's perceptions of living a traumatic childbirth experience and factors related to a birth experience. Int J Environ Res Public Health 2019;16:1654.
137. Roegiers L, Molénat F. Stress et grossesse: Quelle prévention pour quel risque ? Eds *Érès*. Toulouse, France, 2011.
138. Sanders RA, Crozier K. How do informal information sources influence women’s decision-making for birth? A meta-synthesis of qualitative studies. BMC Pregnancy Childbirth 2018;18:21.
139. Simkin P. Birth plans: After 25 years, women still want to be heard. Birth 2007;34:49-51.
140. Smoleniec JS, James DK. Does having a birth plan affect operative delivery rate? J Obstet Gynaecol 1992;12:394-7.
141. Soriano-Vidal FJ, Vila-Candel R, Soriano-Martin PJ, Tejedor-Tornero A, Castro– Sanchez E. The effect of prenatal education classes on the birth expectations of spanish women. Midwifery 2018;60:41–7.
142. Springer D. Birth plans: the effect on anxiety in pregnant women. Int J Childbirth Educ 1996:11:20-5.
143. Suarez-Cortes M, Armero-Barranco D, Canteras-Jordana M, Martinez-Roche ME. Use and influence of delivery and birth plans in the humanizing delivery process. Rev Lat Am Enfermagem 2015;23:520-6.
144. The American College of Nurse Midwives. Share with women. Writing a birth plan. J Midwifery Women’s Health 2014;59:227-8.
145. Thompson R, Miller YD. Birth control: To what extent do women report being informed and involved in decisions about pregnancy and birth procedures? BMC Pregnancy and childbirth 2014;14:62.
146. Too SK. Do birth plans empower women? A study of midwives’views. Nurs Stand1996;10:44-8.
147. Too SK. Do birth plans empower women? A study of their views. Nurs Stand1996;10:33-7.
148. Vander Linden R, Roegiers L. Place du père dans le champ périnatal. Rev Med Perinat 2017;9:241-6.
149. Villarmea S, Kelly B. Barriers to establishing shared decision-making in childbirth: Unveiling epistemic stereotypes about women in labour. J Eval Clin Pract 2020;26:515-9.
150. Waller-Wise R. [Birth plans: Encouraging patient engagement.](https://pubmed.ncbi.nlm.nih.gov/30643368/) J Perinat Educ 2016;25:215-22.
151. Welsh JV, Symon AG. Unique and proforma birth plans: A qualitative exploration of midwives’ experiences. Midwifery 2014;30:885-91.
152. Westergren A, Edin K, Walsh D, Christianson M. Autonomous and dependent - The dichotomy of birth: A feminist analysis of birth pPlans in Sweden. Midwifery 2019;68:56–64.
153. White-Corey S. Birth plans: Ticket to the OR? MCN Am J Matern Child Nurs 2013;38:268-73.
154. White Ribbon Alliance. Respectful maternity care: The Universal Rights of Childbearing Women. Eds *White Ribbon Alliance*. Washington, USA; 2011.
155. Whitford HM, Entwistle VA, van Teijlingen E, et al. Use of a birth plan within woman-held maternity records: A qualitative study with women and staff in northeast Scotland. Birth 2014;41:283-9.
156. Whitford HM, Hillan EM. Women's perceptions of birth plans. Midwifery 1998;14:248-53.
157. World Heath Organization (2006). Standard for maternal and neonatal care. Birth and emergency preparedness in antenatal care. Integrated management of pregnancy and childbirth (IMPAC). 2006 Available at: https://www.who.int/publications/i/item/standards-for-maternal-and-neonatal-care. Accessed August 17, 2023.
158. World Heath Organization (2018). Recommendations. Intrapartum care for a positive childbirth experience. Available at: <https://apps.who.int/iris/bitstream/handle/10665/272447/WHO-RHR-18.12-eng.pdf>. Accessed August 17, 2023.
159. Yam EA, Grossman AA, Goldman LA, Garcia SG. Introducing birth plans in Mexico: An exploratory study in a hospital serving low-income Mexican. Birth 2007;34:42-8.
160. Yuill C, McCourt C, Cheyne H, Leister N. Women’s experiences of decision-making and informed choice about pregnancy and birth care: A systematic review and meta-synthesis of qualitative research. BMC Pregnancy Childbirth 2020;20:343.

**Websites:**

1. How to make a birth plan. National Health Service, England. Available at: <https://www.nhs.uk/pregnancy/labour-and-birth/preparing-for-the-birth/how-to-make-a-birth-plan/> .
2. Developing a birth plan. Department of Health, State Government of Victoria, Australia. Available at: <https://www.betterhealth.vic.gov.au/health/servicesandsupport/developing-a-birth-plan>
3. Writing a birth plan. American College of Nurse-Midwives. Available at: <https://onlinelibrary.wiley.com/doi/pdf/10.1111/jmwh.12192>
4. Chilbirth connection. National Partnership for women and their and families. Available at: <http://www.childbirthconnection.org/>
5. . Scottish Woman Held Maternity Record V6 Nov 2011. Healthcare Improvement Scotland. Available at: <http://www.healthcareimprovementscotland.org/our_work/reproductive,_maternal__child/woman_held_maternity_record/swhmr_maternity_record.aspx>
6. Sample birth plan template. American College of Obstetricians and Gynecologists. Available at: <https://www.acog.org/womens-health/health-tools/sample-birth-plan>
7. Making a birth plan. Department of Health and Aged Care, Australian government. Available at: <https://www.pregnancybirthbaby.org.au/making-a-birth-plan>
8. Birth plan. Institut National de Santé Publique du Québec. Available at: <https://www.inspq.qc.ca/en/tiny-tot/pregnancy/preparing-birth/birth-plan>
9. Birth planning. New Zealand College of Midwives. Available at: <https://www.midwife.org.nz/women/preparing-for-baby/birth-planning/>
10. Writing a birth plan and deciding about pain relief. National Childbirth Trust, England. Available at: https://www.nct.org.uk/pregnancy/dads-be/writing-birth-plan-and-deciding-about-pain-relief

**Supplementary file 2**: Preamble to the structured birth plan intended for the woman and her partner.

The writing of a birth plan has been recommended in France since 2005. Every pregnant woman or parental couple has the right to write such a plan. More than the expression of your child's birth as you've dreamed it, the plan is a tool of communication between parents and professionals to prepare for this birth. Its purpose to build trust between you, your partner or other adult who comes to support you, and the professionals who will be at your side. The idea is “to do it together" to create a cocoon of physical and emotional safety. Knowing you better allows the team to respond better to meet your priority needs, whatever the final outcome of the birth.

You can use the birth plan to envision and discuss the different stages of the delivery and what follows it (labor, birth, care for you and your child, hospitalization at the maternity ward or follow-up at home, etc.). According to the course of your pregnancy and your delivery, you may request to change some of your wishes, and others may not be possible for medical reasons. For that reason it is important to talk about the plan early on and to anticipate the possible scenarios for your follow-up and your delivery. Nonetheless, for any medical procedure concerning either you or your child, the professionals must give you clear, appropriate, and evidence-based information so that you can decide to consent or refuse.

To construct your birth plan, it can be important for you to learn about the practices of the place where you will give birth. For this, you can rely on all of the professionals who are supporting your pregnancy, in particular, during the childbirth and parenting preparation class.

Your birth plan can take whatever form you want (blank paper, a structured paper or computer template or model, or just telling the team what you want, etc.). You can make changes to it throughout your pregnancy and your delivery. This form is one of the possibilities for expressing your wishes and your needs. You can use it however you want: only complete some parts, use it as a basis for discussions, raise other subjects, etc. The essential is to be able to ask all the questions that are important for you, so that you can feel confident about giving birth to your child.
